# *CrystalMELA*: a new crystallographic machine learning platform for crystal system determination

**DOI:** 10.1107/S1600576723000596

**Published:** 2023-02-28

**Authors:** Nicola Corriero, Rosanna Rizzi, Gaetano Settembre, Nicoletta Del Buono, Domenico Diacono

**Affiliations:** a Institute of Crystallography, CNR, Bari, Italy; b Department of Mathematics, University of Bari Aldo Moro, Bari, Italy; c National Institute for Nuclear Physics, Bari, Italy; SLAC National Accelerator Laboratory, Menlo Park, USA

**Keywords:** X-ray diffraction, crystal system determination, machine learning web platform

## Abstract

Determination of the crystal system and space group is the first step of crystal structure analysis. Often this turns out to be a bottleneck in the material characterization workflow for polycrystalline compounds, thus requiring manual interventions. This work proposes a new machine-learning (ML)-based web platform, *CrystalMELA* (Crystallography MachinE LeArning), for crystal systems classification. Two different ML models, random forest and convolutional neural network, are available through the platform, as well as the extremely randomized trees algorithm, available from the literature. The ML models learned from simulated powder X-ray diffraction patterns of more than 280 000 published crystal structures from organic, inorganic and metal–organic compounds and minerals which were collected from the POW_COD database. A crystal system classification accuracy of 70%, which improved to more than 90% when considering the Top-2 classification accuracy, was obtained in tenfold cross-validation. The validity of the trained models has also been tested against independent experimental data of published compounds. The classification options in the *CrystalMELA* platform are powerful, easy to use and supported by a user-friendly graphic interface. They can be extended over time with contributions from the community. The tool is freely available at https://www.ba.ic.cnr.it/softwareic/crystalmela/ following registration.

## Abstract 2

A new artificial-intelligence-based platform, *CrystalMELA*, that can implement machine-learning models has been developed. Powder X-ray diffraction patterns of organic, inorganic and metal–organic compounds and minerals were used to train and test the learning models, and *CrystalMELA* has been employed for crystal system classification.

## 1. Introduction

In the study of new materials, structure characterization is one of the most important tasks because knowledge of the crystalline structure facilitates understanding of material properties (De Graef & McHenry, 2012 ▸). Powder X-ray diffraction (PXRD) is the most appropriate technique to research many materials; the experimental data collected are submitted to powerful crystallographic algorithms, able to perform the necessary steps in the structure solution process, such as indexing, space group determination, integrated intensity estimation, structure solution in reciprocal or direct space, and structure model refinement via the Rietveld method (Rietveld, 1969 ▸). Data from the 3D molecular structures obtained are then stored in various databases, containing either specific classes of materials, like the Inorganic Crystal Structure Database (ICSD; https://www.fiz-karlsruhe.de/icsd.html) and the Cambridge Structural Database (CSD; https://www.ccdc.cam.ac.uk/; Groom *et al.*, 2016 ▸), or a collection of organic, inorganic and metal–organic compounds and minerals, like the commercial Powder Diffraction File (PDF) (ICDD; https://www.icdd.com; Faber & Fawcett, 2002 ▸; Kabekkodu *et al.*, 2002 ▸) and the freely available Crystallography Open Database (COD; https://www.crystallography.net/cod; Graˇzulis *et al.*, 2009 ▸, 2012 ▸).

A PXRD pattern can be affected by peak overlaps, difficulty in background estimation, the presence of preferred orientation effects and limited experimental resolution, which make structure solution non-trivial. Most importantly, can be difficult to perform the critical initial steps such as pattern indexing and space group determination, especially if more than one chemical phase is present in the compound. If the unit cell is incorrectly defined, structure solution may not be possible. These difficulties arise despite the progress, availability and variety (in terms of strategies and methods implemented) of automatic indexing software such as *DICVOL* (Boultif & Louër, 2004 ▸), *N-TREOR09* (Altomare *et al.*, 2009 ▸), *ITO* (Visser, 1969 ▸), *McMaille* (Le Bail, 2004 ▸) and *X-CELL* (Neumann, 2003 ▸).

In the past few years, extraordinary advances in data-driven models and the availability of large amounts of experimental data from many different sources have enabled the development and application of artificial intelligence in materials science (Mueller *et al.*, 2016 ▸; Agrawal & Choudhary, 2016 ▸; Butler *et al.*, 2018 ▸; Schmidt *et al.*, 2019 ▸), especially machine-learning (ML) algorithms for diffraction data analysis. There is widespread literature clearly demonstrating the ability of ML models to make predictions based on correlations found in measured or calculated diffraction data. In terms of crystal system prediction, it is interesting to consider the work of Suzuki *et al.* (2020 ▸), who demonstrated the potential of a simple and fast tree-ensemble-based ML model that manages the PXRD patterns as deconvoluted and discrete peak positions. Other notable contributions are found in the literature (Park *et al.*, 2017 ▸; Do Lee *et al.*, 2022 ▸; Vecsei *et al.*, 2019 ▸; Zaloga *et al.*, 2020 ▸), where deep ML models based on convolutional neural networks (CNNs) were trained on simulated PXRD patterns used as a sort of picture rather than as a set of peak positions and intensities.

Such successful applications have been achieved for a narrow range of specific materials, namely inorganic compounds, generally from the ICSD. This is significantly populated with high-symmetry structures with relatively small lattice parameters (Chitturi *et al.*, 2021 ▸). For thin-film perovskite structures, Oviedo *et al.* (2019 ▸) tested multiple supervised ML approaches coupled with a data augmentation strategy for dimensionality and space group classification, whereas Chakraborty & Sharma (2020 ▸) deployed a variation of a CNN for crystal system classification. Finally, Ziletti *et al.* (2018 ▸) introduced a deep learning neural network model to automatically classify defective structures by crystal symmetry, starting from a set of atomic coordinates and lattice parameters. Similar types of classification analysis also occur in the field of electron diffraction (Aguiar *et al.*, 2019 ▸; Kaufmann *et al.*, 2020 ▸). In addition, ML methods have been applied to tasks such as phase identification (Lee *et al.*, 2020 ▸; Maffettone *et al.*, 2021 ▸).

Stimulated by the ever-increasing number of ML applications for crystallographic data analysis, we propose the ML-based web graphic platform *CrystalMELA* (Crystallography MachinE LeArning). The aim is to overcome the difficulties posed by the structure solution process from PXRD data, and to complement traditional indexing approaches. The tool is currently designed for the classification of the seven crystal classes (triclinic, monoclinic, orthorhombic, tetragonal, trigonal, hexagonal and cubic) and is freely available at https://www.ba.ic.cnr.it/softwareic/crystalmela, following initial registration. The *CrystalMELA* platform is not limited to experts but allows even novices to quickly determine the crystal system for novel compounds. A key strength and original aspect of the present approach is that it advances and supports the process of structure solution, which is essential for providing insights into the properties and functions of a sample under study. This purpose is even more plausible with the planned future extension to many other conventional theoretical rules-based tasks in materials science (*e.g.* determination of cell parameters). The platform can be applied in the case of failure of conventional methods and/or for supporting the results obtained by traditional approaches.

*CrystalMELA* is supported by a user-friendly graphic interface that makes it very easy to use. In the current version, the platform can run three different and complementary ML models: a CNN, an interpretable simple random forest (RF) and an extremely randomized tree (ExRT). The ExRT code is an adaptation of that proposed by Suzuki *et al.* (2020 ▸), accessible in their open GitHub repository.

Unlike specialized models available in the literature, which are typically trained on specific and limited classes of materials (almost exclusively inorganic compounds), another key strength of this study is to make ML models designed to handle different types of compounds more accessible. The algorithms were trained on simulated PXRD patterns (*i.e.* no counting statistics and background, no preferred orientation *etc*.) from more than 280 000 real data of minerals and organic, inorganic and metal–organic compounds selected from the POW_COD database (Altomare *et al.*, 2015 ▸). In addition to the complete data set, the models were trained on two subsets containing only organic (metal–organics are considered organic) and inorganic compounds.

The crystal system classification accuracy achieved in tenfold cross-validation (10CV; https://scikit-learn.org/stable/modules/cross_validation.html) was 70%, which rises to more than 90% when considering Top-2 prediction accuracy (see Section 5 for the meaning of these terms). Similar results were obtained when the performance and efficiency of the trained models were tested on a large set of experimental data belonging to 110 previously published crystal structures. The results are in full agreement with those reported in the literature.

To the best of our knowledge, only two ML-based platforms similar to *CrystalMELA* have been reported in the literature: (1) *CRYSPNet*, designed to predict the crystal system, space group and lattice parameters, based on a combination of several multi-layer perceptron models using the chemical composition of more than 100 000 entries from the ICSD (Liang *et al.*, 2020 ▸). The tool has no graphical interface and can be used from the command line after downloading the project and models from GitHub. (2) *PDFitc*, a cloud-based platform hosting applications for PDF data analysis of crystalline powders and nanomaterials (Yang *et al.*, 2021 ▸).

Compared with other tools, the main innovative aspect of *CrystalMELA* is its ability to use different and complementary ML models, whose simultaneous deployment in the prediction of the crystalline system greatly increases the chances of success. In addition, the platform has the advantage of being updated by following the progressive increase of information stored in the POW_COD database, whose growth can improve the training phase of ML models. *CrystalMELA* is supported by a user-friendly graphic interface, which makes the use of the various available options extremely easy. Finally, it can be extended over time with the implementation of further ML models to perform other crystallographic tasks (*i.e.* space group and lattice parameter prediction). These are in the authors’ future work plan.

## 2. Data preparation

Conventional PXRD patterns (experimental or theoretical) employed in materials research can be used as effective descriptors for ML applications. For training the ML models implemented in the *CrystalMELA* platform, the PXRD patterns used were computed theoretically from the crystal structure solutions stored in POW_COD, an SQLite non-commercial standalone relational database consisting of a collection of entries whose main crystallographic information is generated from the structure information in CIF format of organic, inorganic and metal–organic compounds and minerals contained in the COD. The synthetic diffraction patterns were calculated using the *EXPO* software (Altomare *et al.*, 2013 ▸) via the option to read the structure data in CIF format (in particular, cell parameters, space group, atomic fractional coordinates and displacement parameters). In terms of pattern simulation parameters, a Pearson VII was used as the profile function, the classical Lorentz–polarization correction was adopted and the peak profile was set with fixed mixing parameters, as well as Caglioti parameters (Caglioti *et al.*, 1958 ▸): *U* = *V* = 0 and *W* = FWHM^2^ (FWHM is full width at half-maximum). Preferred orientation was not considered, and the theoretically computed patterns lack the counting statistics and background signals present in real experimental data. The parameters used, which represent the default choices of the *EXPO* software, allow the user to generate plausible PXRD patterns with a good similarity to the real data, thus helping the trained models generalize better to experimental conditions. About 490 000 compounds were extracted, producing the labeled data set {*z*, *l_z_*}, where *z* is the input to the model (the PXRD pattern) and *l_z_* is the correct label (the crystal system to which the pattern belongs). Each compound in the data set is described by the following information:

(1) Diffraction pattern. A set of points (*x_i_, y_i_*), *i* = 1,…, 4501, where *x_i_* and *y_i_* represent the scattering angle 2θ_*i*_ and the corresponding profile intensity value, respectively. The X-ray wavelength was set to 1.54056 Å (Cu *K*α_1_) and the spanned 2θ range focuses on 0–90° to avoid needing to specify a range containing a certain number of peaks. If the 2θ range of the submitted input data is outside of the default choice, it will automatically be cut to 90° if longer or increased to 90° by adding zero to each missing intensity value if shorter. Tests were performed to verify that this choice did not negatively affect the performance of the model. The step size was 0.02° (2θ) and the intensities were normalized such that their largest value was 1000.

(2) Class label representing the crystal system. As the removal of ‘harmful data’ from the training data set is essential to avoid adverse effects on an ML model, we pre-processed the data, excluding compounds that exhibit one of the following issues: (i) Atomic coordinates are not available. (ii) The lattice parameters are large (>40 Å) or small (<2.5 Å); these structures are particularly complex or have few Bragg peaks, respectively. As such, they represent extreme cases and real occurrences are infrequent. The presence of such outliers in the training data could therefore compromise the performance of the ML models. (iii) The weighted profile *R* factor (*R*_wp_) > 10%. A large discrepancy index value implies poor-quality structural refinement.

**Figure 1:**
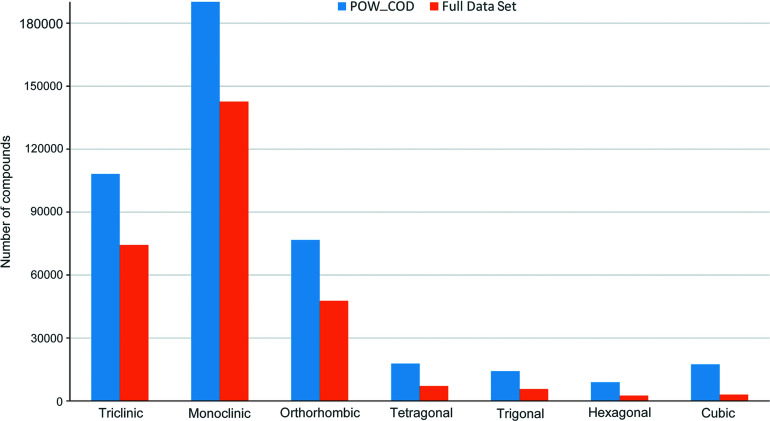
Distribution of samples among the seven crystal systems (*x* axis) in the POW_COD (blue) and full (orange) data sets.

Data manipulation was handled by the *Pandas* (v.1.2.4) (McKinney, 2010 ▸) and *NumPy* (v.1.29.1) (Harris *et al.*, 2020 ▸) packages. After pre-processing, 283 006 entries (hereafter referred to as the full data set) remained and were used to train the models. The distribution of samples among the seven crystal systems in POW_COD and the full data set is shown in Fig. 1 ▸; a large imbalance between the classes is evident.

**Figure 2:**
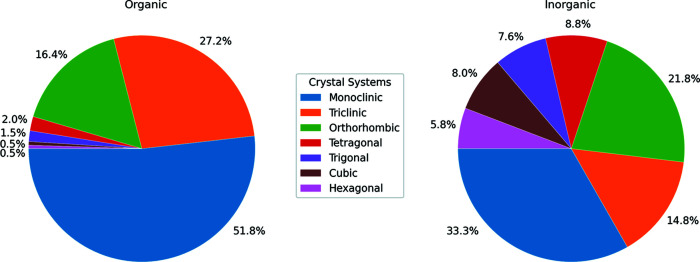
Distribution of crystal systems (class label) in organic and inorganic data sets.

The full data set has been partitioned into two subsets containing organic compounds (herein referred to as the organic data set, with 261 223 entries; metal–organics are considered organic) and inorganic compounds (herein referred to as the inorganic data set, with 21 783 entries), respectively. The split provides the *CrystalMELA* user with the possibility to query a specific data set when the nature of the sample under investigation is known *a priori*, thus sparing computing time. Fig. 2 ▸ reports the distribution of crystal systems in the organic and inorganic data sets. They exhibit complementary distributions which justify the choice to make available the three data sets on *CrystalMELA*. The three algorithms (CNN, RF and ExRT) were trained on all data sets, giving rise to nine final independent models.

## 3. Machine-learning architecture setup

ML constitutes an interesting perspective for tackling the classification problem considered in this study. Different types of supervised learning have been tested (RF, decision tree, *k*-nearest neighbor, support vector machine, naive Bayes, multilayer perceptron and extreme gradient boosting, and CNN) with different impacts in order to derive efficient classification models for the seven crystal systems using PXRD data or features directly computed from them.

The selected ML models constituting the core of *CrystalMELA* are a deep ML based on a CNN, chosen primarily for its ability to automatically extract features from PXRD patterns without the use of any handcrafted feature engineering; an RF model which demonstrates the best performances among the tested classic ML models; and an ExRT model proposed by Suzuki *et al.* (2020 ▸). Significant differences between the performances of the three models are not expected, but rather a synergy derived from their complementarity.

The tenfold cross-validation (10CV) method on the three algorithms was run with the full, organic and inorganic data sets to tune the hyperparameters and evaluate the optimal configuration for each model. The results show no significant differences across the three data sets.

The main characteristics of the models implemented in *CrystalMELA* are described below.

### 3.1. Convolutional neural network model

The 1D CNN employed was trained using the entire diffraction pattern as a 1D input picture. This type of input takes advantage of conventional indexing approaches because it gives the same weight to the low- and high-angle regions of the PXRD pattern. Starting from the Python source code of the CNN model implemented by Park *et al.* (2017 ▸), we tuned the hyperparameters to obtain the maximum performance on the diffraction data sets used. The Adam optimization algorithm (Kingma & Ba, 2015 ▸) with a default learning rate of 0.001 was used.

**Figure 3:**
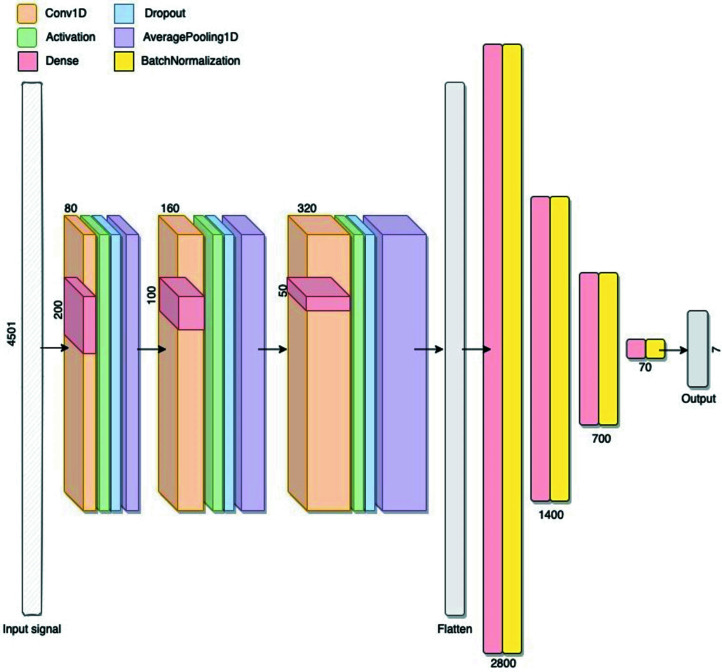
Architecture of the CNN composed of 22 layers: the features include an extraction section, constituted by three convolutional blocks each formed by a Conv1D layer followed by activation, dropout and average pooling layers. The number of Conv1D filters is 80 in the first block and increases incrementally by the same amount in each of the subsequent blocks to become 240 in the last one. The kernel size starts at 200 and is divided by 2 in the second block and by 4 in the third one. Other parameters include sub-sample length = 2, padding = ‘same’ and activation function = ‘relu’. The dropout rate is 0.3 in each block, and the average pooling 1D layers use a pool size of 3. The flattened layer is followed by the classification section, constituted by four densely connected blocks, each formed by a dense layer followed by a batch normalization one. The numbers of neurons used in the dense layer are 2800, 1400, 700 and 70. Each dense layer uses a l2 kernel regularizer and the ‘relu’ activation function, except for the last one which uses ‘tanh’. The last block is followed by the output layer formed of seven units (one for each crystal class), with the ‘softmax’ activation function, to ensure that the sum of the seven output neuron values is always equal to 1.

The final architecture of the CNN is depicted in Fig. 3 ▸. The first layer takes 4501 values as input, each representing a profile intensity value in the 2θ range from 0 to 90°, normalized in the [0, 1] interval. The early stopping approach, with patience = 50 and min delta = 1 × 10^−7^, was adopted to minimize network overfitting, *i.e.* training was stopped at the point when the performance on a validation data set starts to degrade, and at the end of the fitting phase the best CNN weights were restored. The class imbalance affecting each data set has been addressed with the use of a random oversampling of the minority classes.

### 3.2. Random forest model

An RF can be used for both regression and classification tasks; it was also preferred for its advantage in providing an interpretable learning model (Breiman, 2001 ▸). The RF model implemented in *CrystalMELA* has been trained using the minimal number and type of features characterizing a PXRD pattern. According to their relevance, the following features are extracted: (1) The position of the first ten peaks in the lower-angle range. We made this choice because, in real experimental data, the peaks in the low-angle region are less sensitive to small changes in the cell parameters than the higher-angle peaks and there is less overlapping. Consequently, a correct determination of the distinct peak positions, especially for low-symmetry cases, is more likely. (2) The total number of peaks in the 2θ range from 0 to 90°. (3) The 2θ position of the highest intensity peak in the pattern.

For PXRD patterns with fewer than ten peaks in total, the remaining peak position values were set to zero (this choice did not negatively affect the performance of the model). To carry out the peak search on each simulated diffraction pattern, we used the *SciPy* signal processing package (version 1.6.2; Virtanen *et al.*, 2020 ▸) based on the work of Du *et al.* (2006 ▸). The peak detection method used corresponds to the function ‘find peaks’. This takes a 1D array and finds all local maxima by the simple comparison of neighboring values. Some experiments were carried out in order to define a better peak search for the available data. Signal intensities were not thresholded, and peaks of calculated diffraction patterns were taken into account regardless of height, so any peak of the diffraction patterns could possibly serve as an input for ML.

The ‘Gini’ criterion was used to measure the quality of a split, while the number of trees and their maximum depth were set to 250 and 30, respectively, and the minimum samples leaf and the split were set as 1 and 2, respectively.

### 3.3. Extremely randomized trees model

Recently, Suzuki *et al.* (2020 ▸) proposed an interpretable ExRT model for crystal system and space group classification. However, this model uses a different representation of the input data compared with the proposed RF described in Section 3.2 [*i.e.* the first ten peaks and the total number of peaks extracted from the diffraction pattern; see Suzuki *et al.* (2020 ▸) for more details]. The code, available at https://github.com/quantumbeam/xrd-symmetry-prediction, was downloaded and trained on the organic, inorganic and full data sets, and implemented on the *CrystalMELA* platform.

## 4. *CrystalMELA* web platform

The *CrystalMELA* web platform (https://www.ba.ic.cnr.it/softwareic/crystalmela) has been developed to predict the seven crystal systems (triclinic, monoclinic, orthorhombic, tetragonal, trigonal, hexagonal and cubic) when PXRD patterns are used as input data.

*CrystalMELA* is designed to be easy to use, as it is supported by a user-friendly graphic interface and intuitive options to run the ML models. The current version can run CNN, RF and ExRT models.

**Figure 4:**
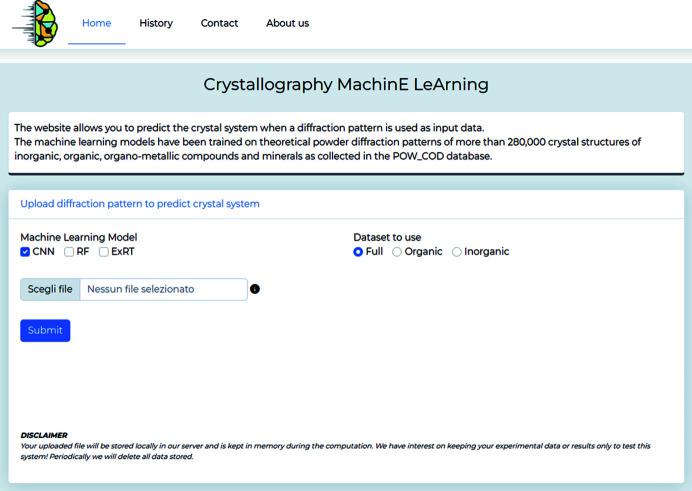
Home web page of the *CrystalMELA* platform.

With respect to similar accessible tools, the availability of several and complementary ML models represents a key strength and novelty of the platform, making it a versatile tool that is generally applicable to all experimental and theoretical PXRD data. It is envisaged that other ML models will be incorporated over time, and multiple tasks will be addressed, such as space group and cell parameter prediction. The *CrystalMELA* home web page is shown in Fig. 4 ▸, and its workflow is described below.

### 4.1. Home page

The function of the Home page is to upload diffraction data and select a number of available options to query the crystal system prediction. Many different PXRD data ASCII files such as XY, DAT, GSAS *etc*. can be imported. The program automatically recognizes the file format by the file name extension (see the platform web page for more details). The options available on the Home page are ‘Machine Learning Model’, which option allows the selection of one or more ML models among the three available in the current version for the crystal system classification, and ‘Dataset to use’, which allows the user to select the data set for the crystal system classification that can be performed on the full data set (default choice) or optionally under the restraining condition on the organic or inorganic data sets.

### 4.2. Results page

After the diffraction data have been loaded, the probability for each of the seven crystal systems is predicted under the conditions set by the user on the Home page. The results are presented as a histogram plot. As an example, if all three ML models are employed at the same time in the Home page, each will return its own evaluated probability bar for each crystal system.

**Figure 5:**
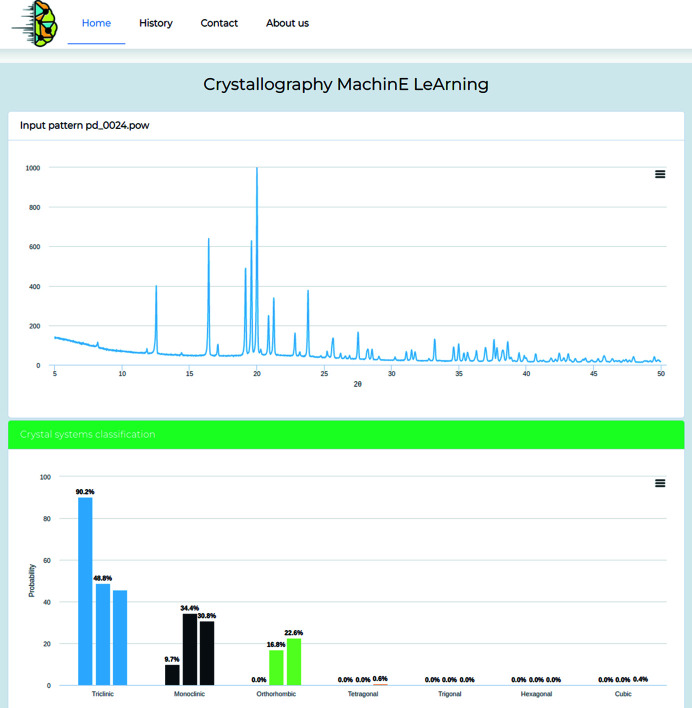
Results page. Input diffraction pattern and crystal systems classification report.

Fig. 5 ▸ is an example of a Results page, showing the input diffraction pattern and the histogram of the predicted probabilities returned by the *CrystalMELA* web platform.

### 4.3. History page

**Figure 6:**
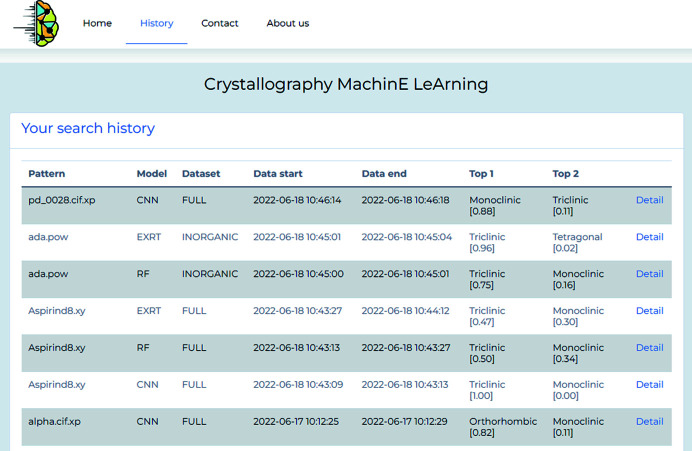
History page.

The History page stores all user sessions. For each, the name of the input data that have already been analyzed, the ML model(s) used, the data set(s) queried, and the obtained Top-1 and Top-2 prediction accuracies (see Section 5 for a definition) are displayed. Fig. 6 ▸ shows an example History page. Users can also review the loaded input diffraction pattern and the histogram of the results by clicking on the Detail link.

### 4.4. Contact

*CystalMELA* is work in progress. The authors encourage interested users to provide suggestions and comments via the Contact page.

### 4.5. Implementation and availability

*CrystalMELA* was created using PHP and Laravel for the backend platform, while PHP, *Bootstrap* (https://getbootstrap.com) and *HighCharts* (https://www.highcharts.com) were used for the frontend. The template is fully responsive and optimized for mobile devices. A relational database was created using *MySQL* (https://www.mysql.com/) to store all runs for statistical information. The software/library names and versions used in the current version (June 2022) of *CrystalMELA* are *EXPO* (version *EXPO2014*), COD svn (revision 212659), *Bootstrap* (version 5.50) and *Highcharts* (version v10.1.0).

## 5. Metrics

Predicting the crystal system is a multi-class classification problem. To evaluate the model performances, the classification accuracy, Top-2 accuracy, *F*1 score and confusion matrix were used as metrics. The canonical accuracy is defined in terms of the number of true positive (TP), true negative (TN), false positive (FP) and false negative (FN) predictions, as follows:

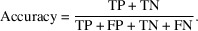

The *F*1 score is given by

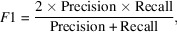

with

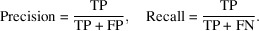

The *F*1 score is the harmonic mean of precision and recall and faciliatates a convenient way to provide a high level of comparison for the classification performance of each model. The computed *F*1 score is ‘weighted’; it is calculated by taking the mean of all per-class *F*1 scores while considering each class support. Concerning the top-*k* accuracy with *k* ≥ 2, it is often used for multi-class classification tasks because the canonical accuracy can be too stringent, especially if the probabilities for several classes are close, in which case all of them are of interest. Specifically, Top-2 accuracy measures the proportion of correct predictions in the two classes with the highest predicted probability.

## 6. Results and discussion

The analysis of the results obtained for the theoretical diffraction data was carried out using the metrics reported in Table 1 ▸ for each ML model when applied to the three data sets over the tenfold cross-validation. The standard deviations over CV are reported in parentheses.

The CNN reached the highest classification accuracy of about 70% with a Top-2 accuracy value of over 90% on all the data sets. For this reason, CNN has been chosen as default model in *CrystalMELA*. The results obtained are comparable to those reported in the literature. Due to the scarcity of inorganic data (21 783 entries) with respect to organic data (261 223 entries), similar accuracy values were obtained on the full and organic data sets. Despite the fact that the inorganic data set is the smallest, it is better classified by all the ML models.

The performances in terms of precision, recall and *F*1 score achieved by each model on the three data sets and specified for each crystal system are reported below.

### 6.1. CNN Results

Table 2 ▸ summarizes the metric values obtained by CNN. For the inorganic and organic data sets the performances are uneven between the classes. To overcome the large imbalance clearly evident among the classes of organic compounds (see Fig. 2 ▸), a random oversample of the minority classes was performed. However, the undersampling of the monoclinic class did not improve the performance of the CNN model. Finally, as expected, for the full data set, the addition of the inorganic compounds does not change the classification performance.

### 6.2. RF results

Table 3 ▸ shows the results obtained by the RF model on the three data sets. The distribution of *F*1 score values for each data set reflects the behavior observed for the CNN model, even though the performance values are slightly lower (particularly in orthorhombic and tetragonal classes for the organic data set).

### 6.3. ExRT results

Table 4 ▸ summarizes the metric results obtained using the ExRT model. The distribution of *F*1 score values for each data set is comparable to that obtained with the RF model, and lower than that given by the CNN model.

We point out that the performances of ExTR differ from those published by Suzuki *et al.* (2020 ▸), where the model was trained using a different data set which directly provides the input features the model requires [downloaded from the reference page reported by Suzuki *et al.* (2020 ▸)]. Generally, the behavior of any data-driven model is strongly dependent on the data set characteristics it is trained on.

## 7. Case studies with real experimental data

To assess the validity of our models on the crystal system classification task, we use real experimental data; these are significantly different from the data sets used to train the models (simulated patterns) in *CrystalMELA*. Although the three ML models are not fully trained to overcome all the problems that real data present (*i.e.* overlapping peaks, noise/background *etc*.), we test their efficiency in classifying real diffraction patterns. The real data set contains crystalline samples from a large PXRD database of already published structures that belong to the private data collections of some of the authors. It consists of 110 diffraction patterns from organic, inorganic and metal–organic compounds of different structural complexity and data quality (using a conventional X-ray diffractometer, and synchrotron and neutron radiation). The distribution of the available real data among the seven crystal systems is reported in Table 5 ▸. The data set is strongly unbalanced: it does not contain any sample belonging to the cubic system, while the hexagonal, tetragonal and trigonal classes amount to 5% of samples. Note that this can heavily compromise the correct classification by the model.

**Figure 7:**
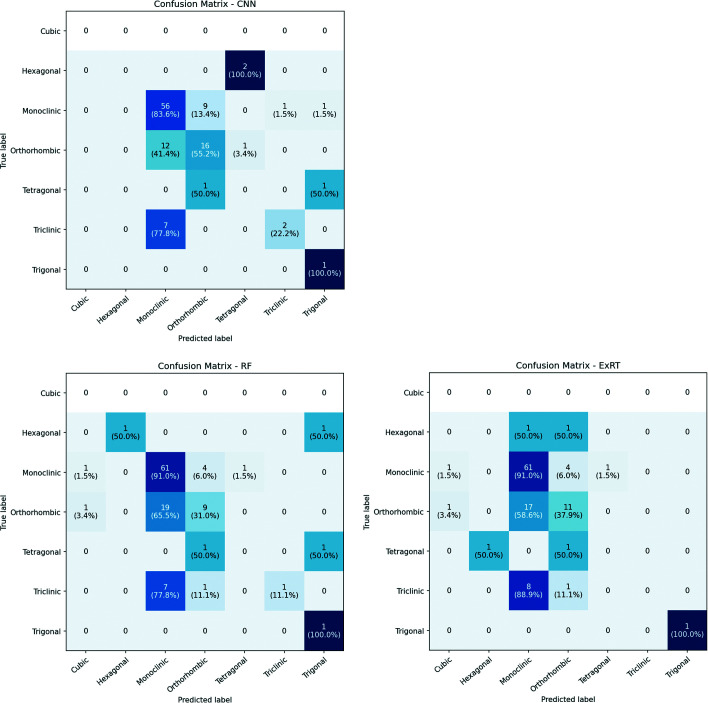
Confusion matrices visualizing and summarizing the performance of the three classification models in *CrystalMELA* on the experimental data set.

To evaluate the capacity of our models to classify the crystal systems when an experimental data set is used, the confusion matrix was evaluated to visualize and summarize the performance of the three models (trained on the full data set). They are reported in Fig. 7 ▸. The elements in the confusion matrix indicate the number of samples correctly (diagonal) and incorrectly classified and their percentages (values in parentheses). All the classification models correctly classify the single sample belonging to the trigonal system, but they were not able to properly identify the two tetragonal samples. The models also present very similar results for the classification of monoclinic samples. As expected, the ML models in *CrystalMELA* are clearly more accurate in classifying the theoretical data sets (with no noise and background like in the training data) than the experimental ones (which contain noise and background not present in the training data). This is the major obstacle in achieving a higher classification accuracy on the experimental data.

A dummy classifier model, which makes predictions without trying to find patterns in the data, has also been added, serving to establish a simple baseline to compare against other more complex classifiers and to calculate metrics on the test set of real data. It can use three different strategies: stratified, uniform and most frequent, the latter was adopted in the present study. Table 6 ▸ reports the performance metrics on the real experimental data set. Accuracy, Top-2 accuracy, balanced accuracy and ‘weighted’ *F*1 score are reported for all models in *CrystalMELA* and for the best dummy classifier. The balanced accuracy in multi-class classification is defined as the average recall obtained on each class to avoid inflated performance estimates on imbalanced data sets. The results obtained are comparable to those obtained for theoretical data sets, demonstrating the validity of the proposed ML models for imperfect data. As mentioned above, one limitation to the success of the ML models when applied to experimental diffraction data is the lack of noise and background signals in the theoretical patterns. A model trained using only such data may interpret the experimental noise as Bragg peaks and potentially cause a misclassification.

## 8. Conclusions

The community ML web platform *CrystalMELA* is designed to provide an easy-to-use and versatile tool for predicting the most likely crystal system of organic, inorganic and metal–organic compounds and minerals. *CrystalMELA* is freely available at https://www.ba.ic.cnr.it/softwareic/crystalmela/. It is envisaged that the platform will host an increasing number of web services over time, but the current version can run CNN, RF and ExRT models, trained on about 280 000 compounds extracted from the POW_COD database. The user can easily upload PXRD data on the platform, querying one or more of the available analysis applications to receive the required crystal system prediction. A good level of prediction accuracy is reached by all the models both on theoretical and on real data, strongly supporting the ability of data-driven algorithms to discover unrecognized characteristics embedded in the experimental data and hidden from the human eye.

## Figures and Tables

**Table 1:** Classification report on a tenfold CV For each ML model the accuracy, Top-2 accuracy and *F*1 score on the inorganic, organic and full data sets are reported.

	Accuracy	Top-2 accuracy	*F*1 score
Inorganic data set			
CNN	0.702 (0.015)	0.894 (0.009)	0.702 (0.015)
RF	0.709 (0.010)	0.894 (0.004)	0.704 (0.011)
ExRT	0.713 (0.008)	0.894 (0.005)	0.710 (0.008)

Organic data set			
CNN	0.676 (0.002)	0.942 (0.003)	0.668 (0.008)
RF	0.624 (0.003)	0.897 (0.001)	0.593 (0.003)
ExRT	0.622 (0.003)	0.894 (0.001)	0.593 (0.003)

Full data set			
CNN	0.666 (0.026)	0.930 (0.026)	0.660 (0.024)
RF	0.619 (0.002)	0.892 (0.001)	0.590 (0.002)
ExRT	0.616 (0.003)	0.888 (0.002)	0.588 (0.003)

**Table 2:** Performances of the CNN model on the inorganic, organic and full data sets For each crystal system the precision, recall and *F*1 score values are reported.

	Triclinic	Monoclinic	Orthorhombic	Tetragonal	Trigonal	Hexagonal	Cubic
Inorganic data set							
Precision	0.66	0.66	0.68	0.72	0.75	0.78	0.95
Recall	0.51	0.76	0.66	0.71	0.70	0.72	0.94
*F*1 score	0.58	0.71	0.67	0.71	0.72	0.75	0.94
	
Organic data set							
Precision	0.71	0.67	0.63	0.63	0.64	0.83	0.91
Recall	0.54	0.82	0.48	0.53	0.66	0.45	0.91
*F*1 score	0.61	0.74	0.54	0.58	0.65	0.58	0.91

Full data set							
Precision	0.70	0.67	0.61	0.57	0.50	0.72	0.93
Recall	0.54	0.80	0.47	0.52	0.70	0.55	0.96
*F*1 score	0.60	0.73	0.53	0.54	0.58	0.62	0.94

**Table 3:** Performances of the RF model on the inorganic, organic and full data sets For each crystal system the precision, recall and *F*1 score values are reported.

	Triclinic	Monoclinic	Orthorhombic	Tetragonal	Trigonal	Hexagonal	Cubic
Inorganic data set							
Precision	0.659	0.640	0.706	0.790	0.766	0.779	0.878
Recall	0.440	0.819	0.643	0.658	0.704	0.684	0.919
*F*1 score	0.527	0.719	0.673	0.718	0.734	0.728	0.898

Organic data set							
Precision	0.629	0.612	0.666	0.691	0.680	0.843	0.875
Recall	0.435	0.860	0.222	0.354	0.504	0.399	0.789
*F*1 score	0.514	0.715	0.333	0.468	0.579	0.523	0.830

Full data set							
Precision	0.633	0.613	0.703	0.741	0.681	0.856	0.915
Recall	0.433	0.864	0.262	0.427	0.575	0.525	0.892
*F*1 score	0.514	0.717	0.381	0.542	0.623	0.650	0.903

**Table 4:** Performances of the ExRT model on the inorganic, organic and full data sets For each crystal system the precision, recall and *F*1 score values are reported.

	Triclinic	Monoclinic	Orthorhombic	Tetragonal	Trigonal	Hexagonal	Cubic
Inorganic data set							
Precision	0.648	0.640	0.707	0.808	0.761	0.782	0.895
Recall	0.460	0.798	0.652	0.650	0.722	0.723	0.934
*F*1 score	0.538	0.711	0.679	0.721	0.740	0.752	0.914

Organic data set							
Precision	0.625	0.611	0.650	0.665	0.652	0.759	0.850
Recall	0.422	0.858	0.239	0.332	0.501	0.395	0.812
*F*1 score	0.504	0.714	0.350	0.443	0.566	0.519	0.831

Full data set							
Precision	0.625	0.612	0.673	0.738	0.685	0.821	0.924
Recall	0.421	0.856	0.280	0.441	0.571	0.548	0.897
*F*1 score	0.503	0.714	0.396	0.552	0.623	0.657	0.910

**Table 5:** Distribution of the real samples among the seven crystal systems

Crystal system	Number of samples	Percentage of total samples (%)
Monoclinic	67	61
Orthorhombic	29	26
Triclinic	9	8
Hexagonal	2	2
Tetragonal	2	2
Trigonal	1	1
Cubic	0	0

**Table 6:** Performances of the models on the real experimental data set

	Accuracy	Top-2 accuracy	Balance accuracy	*F*1 score
CNN	0.682	0.936	0.435	0.666
RF	0.664	0.873	0.472	0.623
ExRT	0.664	0.855	0.382	0.615
Dummy	0.610	0.620	0.170	0.460

## References

[bb1] Agrawal, A. & Choudhary, A. (2016). *APL Mater.* **4**, 053208.

[bb2] Aguiar, J., Gong, M., Unocic, R., Tasdizen, T. & Miller, B. (2019). *Sci. Adv.* **5**, eaaw1949. 10.1126/sciadv.aaw1949 PMC6957330 31976364

[bb3] Altomare, A., Campi, G., Cuocci, C., Eriksson, L., Giacovazzo, C., Moliterni, A., Rizzi, R. & Werner, P.-E. (2009). *J. Appl. Cryst.* **42**, 768–775.

[bb4] Altomare, A., Corriero, N., Cuocci, C., Falcicchio, A., Moliterni, A. & Rizzi, R. (2015). *J. Appl. Cryst.* **48**, 598–603.

[bb5] Altomare, A., Cuocci, C., Giacovazzo, C., Moliterni, A., Rizzi, R., Corriero, N. & Falcicchio, A. (2013). *J. Appl. Cryst.* **46**, 1231–1235.

[bb6] Boultif, A. & Louër, D. (2004). *J. Appl. Cryst.* **37**, 724–731.

[bb7] Breiman, L. (2001). *Mach. Learn.* **45**, 5–32.

[bb8] Butler, K., Davies, D., Cartwright, H., Isayev, O. & Walsh, A. (2018). *Nature*, **559**, 547–555. 10.1038/s41586-018-0337-2 30046072

[bb101] Caglioti, G., Paoletti, A. & Ricci, F. P. (1958). *Nucl. Instrum.* **3**, 223–228.

[bb9] Chakraborty, A. & Sharma, R. (2020). *2020 International Conference on CyberWorlds*, 29 September–1 October 2020, Caen, France, pp. 49–54. IEEE.

[bb10] Chitturi, S. R., Ratner, D., Walroth, R. C., Thampy, V., Reed, E. J., Dunne, M., Tassone, C. J. & Stone, K. H. (2021). *J. Appl. Cryst.* **54**, 1799–1810. 10.1107/S1600576721010840 PMC8662964 34963768

[bb11] De Graef, M. & McHenry, M. (2012). *Structure of Materials: an Introduction to Crystallography, Diffraction and Symmetry.* Cambridge University Press.

[bb12] Do Lee, B., Lee, J.-W., Park, W. B., Park, J., Cho, M.-Y., Singh, S. P., Pyo, M. & Sohn, K.-S. (2022). *Adv. Intell. Syst.* **4**, 1–13.

[bb13] Du, P., Kibbe, W. A. & Lin, S. M. (2006). *Bioinformatics*, **22**, 2059–2065. 10.1093/bioinformatics/btl355 16820428

[bb14] Faber, J. & Fawcett, T. (2002). *Acta Cryst.* B**58**, 325–332. 10.1107/s0108768102003312 12037351

[bb15] Gražulis, S., Chateigner, D., Downs, R. T., Yokochi, A. F. T., Quirós, M., Lutterotti, L., Manakova, E., Butkus, J., Moeck, P. & Le Bail, A. (2009). *J. Appl. Cryst.* **42**, 726–729. 10.1107/S0021889809016690 PMC3253730 22477773

[bb16] Gražulis, S., Daškevič, A., Merkys, A., Chateigner, D., Lutterotti, L., Quiŕos, M., Serebryanaya, N., Moeck, P., Downs, R. & Le Bail, A. (2012). *Nucleic Acids Res.* **40**, D420–D427. 10.1093/nar/gkr900 PMC3245043 22070882

[bb40] Groom, C. R., Bruno, I. J., Lightfoot, M. P. & Ward, S. C. (2016). *Acta Cryst.* B**72**, 171–179. 10.1107/S2052520616003954 PMC4822653 27048719

[bb17] Harris, C. R., Millman, K. J., van der Walt, S. J., Gommers, R., Virtanen, P., Cournapeau, D., Wieser, E., Taylor, J., Berg, S., Smith, N. J., Kern, R., Picus, M., Hoyer, S., van Kerkwijk, M. H., Brett, M., Haldane, A., del Río, J. F., Wiebe, M., Peterson, P., Gérard-Marchant, P., Sheppard, K., Reddy, T., Weckesser, W., Abbasi, H., Gohlke, C. & Oliphant, T. E. (2020). *Nature*, **585**, 357–362. 10.1038/s41586-020-2649-2 PMC7759461 32939066

[bb18] Kabekkodu, S. N., Faber, J. & Fawcett, T. (2002). *Acta Cryst.* B**58**, 333–337. 10.1107/s0108768102002458 12037352

[bb19] Kaufmann, K., Zhu, C., Rosengarten, A., Maryanovsky, D., Harrington, T., Marin, E. & Vecchio, K. (2020). *Science*, **367**, 564–568. 10.1126/science.aay3062 32001653

[bb20] Kingma, D. P. & Ba, J. (2015). *arXiv*:1412.6980 [cs.LG].

[bb21] Le Bail, A. (2004). *Powder Diffr.* **19**, 249–254.

[bb22] Lee, J.-W., Park, W. B., Lee, J. H., Singh, S. P. & Sohn, K.-S. A. (2020). *Nat. Commun.* **11**, 86. 10.1038/s41467-019-13749-3 PMC6941984 31900391

[bb23] Liang, H., Stanev, V., Kusne, G. & Takeuchi, I. (2020). *Phys. Rev. Mater.* **4**, 123802.

[bb24] Maffettone, P., Banko, L., Cui, P., Lysogorskiy, Y., Little, M. A., Olds, D., Ludwig, A. & Cooper, A. I. (2021). *Nat. Comput. Sci.* **1**, 290–297. 10.1038/s43588-021-00059-2 38217168

[bb25] McKinney, W. (2010). *SciPy2010. Proceedings of the 9th Python in Science Conference*, 28 June–3 July, Austin, Texas, USA, edited by S. van der Walt & J. Millman, pp. 56–61.

[bb26] Mueller, T., Kusne, A. G. & Ramprasad, R. (2016). *Rev. Comput. Chem.* **4**, 186–273.

[bb27] Neumann, M. A. (2003). *J. Appl. Cryst.* **36**, 356–365.

[bb28] Oviedo, F., Ren, Z., Sun, S., Settens, C. M., Liu, Z., Hartono, N. T. P., Ramasamy, S., DeCost, B. L., Tian, S. I. P., Romano, G., Kusne, A. G. & Buonassisi, T. (2019). *NPJ Comput. Mater.* **5**, 60.

[bb29] Park, W. B., Chung, J., Jung, J., Sohn, K., Singh, S. P., Pyo, M., Shin, N. & Sohn, K.-S. (2017). *IUCrJ*, **4**, 486–494. 10.1107/S205225251700714X PMC5571811 28875035

[bb30] Rietveld, H. M. (1969). *J. Appl. Cryst.* **2**, 65–71.

[bb31] Schmidt, J., Marques, M. R., Botti, S. & Marques, M. A. L. (2019). *NPJ Comput. Mater.* **5**, 83.

[bb32] Suzuki, Y., Hino, H., Hawai, T., Saito, K., Kotsugi, M. & Ono, K. (2020). *Sci. Rep.* **10**, 21790. 10.1038/s41598-020-77474-4 PMC7732852 33311555

[bb34] Vecsei, P. M., Choo, K., Chang, J. & Neupert, T. (2019). *Phys. Rev. B*, **99**, 245120.

[bb35] Virtanen, P., Gommers, R., Oliphant, T. E., Haberland, M., Reddy, T., Cournapeau, D., Burovski, E., Peterson, P., Weckesser, W., Bright, J., van der Walt, S. J., Brett, M., Wilson, J., Millman, K. J., Mayorov, N., Nelson, A. R. J., Jones, E., Kern, R., Larson, E., Carey, C. J., Polat, İ., Feng, Y., Moore, E. W., VanderPlas, J., Laxalde, D., Perktold, J., Cimrman, R., Henriksen, I., Quintero, E. A., Harris, C. R., Archibald, A. M., Ribeiro, A. H., Pedregosa, F., van Mulbregt, P., Vijaykumar, A., Bardelli, A. P., Rothberg, A., Hilboll, A., Kloeckner, A., Scopatz, A., Lee, A., Rokem, A., Woods, C. N., Fulton, C., Masson, C., Häggström, C., Fitzgerald, C., Nicholson, D. A., Hagen, D. R., Pasechnik, D. V., Olivetti, E., Martin, E., Wieser, E., Silva, F., Lenders, F., Wilhelm, F., Young, G., Price, G. A., Ingold, G., Allen, G. E., Lee, G. R., Audren, H., Probst, I., Dietrich, J. P., Silterra, J., Webber, J. T., Slavič, J., Nothman, J., Buchner, J., Kulick, J., Schönberger, J. L., de Miranda Cardoso, J. V., Reimer, J., Harrington, J., Rodríguez, J. L. C., Nunez-Iglesias, J., Kuczynski, J., Tritz, K., Thoma, M., Newville, M., Kümmerer, M., Bolingbroke, M., Tartre, M., Pak, M., Smith, N. J., Nowaczyk, N., Shebanov, N., Pavlyk, O., Brodtkorb, P. A., Lee, P., McGibbon, R. T., Feldbauer, R., Lewis, S., Tygier, S., Sievert, S., Vigna, S., Peterson, S., More, S., Pudlik, T., Oshima, T., Pingel, T. J., Robitaille, T. P., Spura, T., Jones, T. R., Cera, T., Leslie, T., Zito, T., Krauss, T., Upadhyay, U., Halchenko, Y. O. & Vázquez-Baeza, Y. (2020). *Nat. Methods*, **17**, 261–272.

[bb36] Visser, J. W. (1969). *J. Appl. Cryst.* **2**, 89–95.

[bb37] Yang, L., Culbertson, E. A., Thomas, N. K., Vuong, H. T., Kjær, E. T. S., Jensen, K. M. Ø., Tuckerd, M. G. & Billinge, S. J. L. (2021). *Acta Cryst.* A**77**, 2–6. 10.1107/S2053273320013066 PMC7842210 33399126

[bb38] Zaloga, A. N., Stanovov, V. V., Bezrukova, O. E., Dubinin, P. S. & Yakimov, I. S. (2020). *Mater. Today Commun.* **25**, 101662.

[bb39] Ziletti, A., Kumar, D., Scheffler, M. & Ghiringhelli, L. (2018). *Nat. Commun.* **9**, 2775. 10.1038/s41467-018-05169-6 PMC6050314 30018362

